# Superparamagnetic Bifunctional Bisphosphonates Nanoparticles: A Potential MRI Contrast Agent for Osteoporosis Therapy and Diagnostic

**DOI:** 10.4061/2010/747852

**Published:** 2010-06-15

**Authors:** Y. Lalatonne, M. Monteil, H. Jouni, J. M. Serfaty, O. Sainte-Catherine, N. Lièvre, S. Kusmia, P. Weinmann, M. Lecouvey, L. Motte

**Affiliations:** ^1^Laboratoire CSPBAT, C2B, FRE 3043 CNRS, Université Paris 13, 93017 Bobigny Cedex, France; ^2^Service de Médecine Nucléaire, Hôpital Avicenne, APHP, 93009 Bobigny Cedex, France; ^3^Service de Radiologie, Hôpital Bichat, APHP, U 698 ISERM, Université Paris 7, 75877 Paris Cedex 18, France; ^4^UPRES 3410 Biothérapies Bénéfices et Risques, Université Paris 13, 93017 Bobigny Cedex, France; ^5^Plateforme d'IRM du Petit Animal, U 970 INSERM, Université Paris 5, 75908 Paris Cedex 10, France

## Abstract

A bone targeting nanosystem is reported here which combined magnetic contrast agent for Magnetic Resonance Imaging (MRI) and a therapeutic agent (bisphosphonates) into one drug delivery system. This new targeting nanoplatform consists of superparamagnetic *γ*Fe_2_O_3_ nanoparticles conjugated to 1,5-dihydroxy-1,5,5-tris-phosphono-pentyl-phosphonic acid (di-HMBPs) molecules with a bisphosphonate function at the outer of the nanoparticle surface for bone targeting. The as-synthesized nanoparticles were evaluated as a specific MRI contrast agent by adsorption study onto hydroxyapatite and MRI measurment. The strong adsorption of the bisphosphonates nanoparticles to hydroxyapatite and their use as MRI T2^∗^ contrast agent were demonstrated. Cellular tests performed on human osteosarcoma cells (MG63) show that *γ*Fe_2_O_3_@di-HMBP hybrid nanomaterial has no citoxity effect in cell viability and may act as a diagnostic and therapeutic system.

## 1. Introduction

**Scheme 1:**
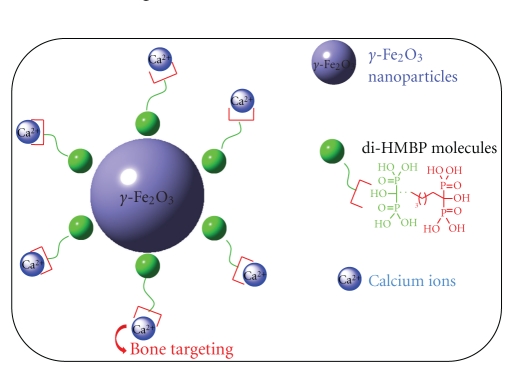
Superparamagnetic *γ*Fe_2_O_3_@di-HMBPs for bone targeting.

Bisphosphonates exhibits a powerful binding affinity to bones and are routinely used for treatment in bone resorption and other bone disorders like Paget's disease, osteoporosis, or tumor induced osteolysis [1]. The binding to bone mineral depends upon the P-C-P structure and is enhanced by including a hydroxyl group (hydroxy methylene bisphosphonate, called HMBP in the text). This was probably due to tridendate binding hydroxyl substituted bisphosphonates to calcium. In contrast, bisphosphonates lacking a hydroxyl group, that provide a bidendate binding to calcium crystals, had significantly lower binding affinities [2]. Hence HMBP molecules, such as Alendronate (4-amino-1-hydroxybutylidene bisphosphonic acid), inhibit osteoclast-mediated bone resorption [3]. With the recent developments in magnetic resonance, in vivo studies showed that patients with, and without, osteoroporotic fractures could better be separated with parameters of bone architecture obtained by MRI than BDM [4]. For molecular imaging, the use of nanoparticles emerge as very exiting nanoobjects in that many functionalities can be added to the surface of the particle. More specifically, superparamagnetic iron oxide [5] (SPIO, hydrodynamic diameter >50 nm) and ultrasmall superparamagnetic iron oxide (USPIO, hydrodynamic diameter <50 nm) particles have been introduced as an MRI contrast agent after the gadolinium chelates and appear to be currently a more relevant agent than Gd chelates due to the high MR signal per unit of metal. As these particles are made of thousands iron atoms, they defeat the inherent low contrast agent sensitivity of MRI and thus can be detected at micromolar concentration of iron. Moreover the iron ions are much less toxic than the gadolinium ones and can be reused or recycled by cells using normal biochemical pathways for iron metabolism [6, 7]. Our previous studies have shown that bisphosphonate such as 1-phenyl-1-hydroxymethylene-1,1-phosphonic acid (HMBP-COOH) [8] or 1-hydroxy-2-(imidazol-1-yl)ethylidene-1,1-bisphosphonic acid (zoledronate) [9] act as very efficient ligand for iron oxide nanoparticles. In the case of quaternary ammonium bisphosphonate coated iron oxide nanocrystals, it has been shown that this hybrid nanocrystals [10] presented adequate performance for blood remanance and weak liver capture. No significant desorption of the coating molecules was observed on steel plates. In recent work [11] it has been demonstrated that pretreatment of metal alloy surface with an aqueous polyallylamine bisphosphonate solution (BP-…NH_2_) result in the formation of a molecular bisphophonate layer that permit the attachment via the amine terminated function of vector binding agent for therapeutic gene delivery. After 30 day incubation, the layer is not altered indicating that a mechanism of desorption reabsorption of BP molecules seems to be highly unlikely. In this article, an innovative approach is presented, leading to the optimization of the nanoparticle structure to achieve selective targeting for osteoporosis imaging and therapy. Superparamagnetic nanoparticle surface are passivated using a bifunctional passivating agent such as 1,5-dihydroxy-1,5,5-tris-phosphono-pentyl-phosphonic acid (call di-HMBP in the text, Scheme 1). One HMBP function complexes the nanocrystal surface and the other one at the outer surface allows bone targeting. A stable ferrofluid (*γ*Fe_2_O_3_@di-HMBP) is obtained on large concentration and pH range. The large numbers of HMBP functionalities on the magnetic core of the particle have a strong affinity for hydroxyapatite and can be used for bone targeting. The feasibility of such process is demonstrated by the complexation of the hybrid nanomaterial to calcium ions and hydroxyapatite and imaged using MRI.

## 2. Materials and Methods

### 2.1. Materials and Reagent

IR spectra were recorded on a Thermo Electron Corporation Nicolet 380 FTIR (KBr pellet). UV-visible spectra were recorded on a Varian Cary 50 Scan UV-Visible spectrophotometer. Transmission electron microscopy (TEM) measurements were carried out using a Philips CM10. ^1^H-NMR spectra were obtained on a Varian Gemini spectrometer at 200 MHz with chemical shifts being reported as ppm from trimethylsilane as internal standard. The size and the zeta potential of the nanocomplex were determined by dynamic laser light scattering (DLS) on a Nano-ZS (Red Badge) ZEN 3600 device (Malvern Instruments, Malvern, UK. All chemicals products used for nanoparticles and bisphosphonate molecules were purchased from Sigma-Aldrich (St Louis, MO). Millipore H_2_O was employed for the preparation of all aqueous solutions.

### 2.2. Synthesis of (1,5-Dihydroxy-1,5,5-Tris-Phosphono-Pentyl)-Phosphonic Acid [12] (Di-HMBPs)

In a 50 mL round-bottom three-neck flask equipped with a thermometer, glutaryl chloride (18 mmol) was added dropwise, under argon, at −5°C, to tris(trimethylsilyl) phosphite (72 mmol). When addition was completed, reaction mixture was allowed to stand at room temperature for 1 hour. The evolution of the reaction was monitored by ^31^P{1H} NMR. Then, volatile fractions were evaporated under reduced pressure (0.1 Torr) before methanolysis (20 mL). After evaporation, crude products were precipitated in diethylether and lyophilized. The pure product was obtained in 95% yield. ^31^P NMR {^1^H} (161.9 MHz, D_2_O) *δ* 19.3, ^1^H NMR (400.1 MHz, D_2_O) *δ* 1.78–2.05 (m, 6H, C(OH)-(CH_2_)_3_-C(OH)), ^13^C NMR {^1^H} (100.6 MHz, D_2_O) *δ* 18.1 (-CH_2_-CH_2_-CH_2_-), 34.0 (-CH_2_-CH_2_-CH_2_-), 73.2 (t, ^1^ *J*_P−C_ = 143.7 Hz, P-C(OH)-P).

### 2.3. Synthesis of *γ* *F* *e*_2_ *O*_3_@Di-HMBP Nanocrystals

To prepare noncoated *γ*Fe_2_O_3_ particles, the first step is to add a solution of dimethylamine 40% in water ((CH_3_)_2_NH, 10.5 mL) to an aqueous micellar solution of ferrous dodecyl sulfate (Fe(DS)_2_) (0.61 g, 10^−3^ mol). The solution is stirred vigorously for 2 hours at 28.5°C and the resulting precipitate of uncoated nanocrystals is isolated from the supernatant by centrifugation. In the second step, this precipitate is washed with an acidic solution (HCl 10^−1^ mol · L^−1^) and a solution of di-HMBPs molecules (*n* = 10^−4^ mol in 30 mL of water) is added. The solution is stirred for two hours at room temperature. The precipitate that appears is washed with an acidic solution (HCl 10^−1^ mol · L^−1^). Free HMBP are isolated from the coated particles thanks to a magnetic field and by centrifugation. The magnetic nanocrystals coated with di-HMBP molecules are dispersed in water. The initial pH is equal to 4 and then progressively increased to pH 7.4 by addition of sodium hydroxide NaOH (10^−1^ mol · L^−1^). The iron concentration is deduced from UV-vis absorption.

### 2.4. Nanocrystal Surface Characterization

FTIR spectroscopy is used to demonstrate nanocrystal surface complexation via phosphonate groups. The average number of molecules per nanocrystal is deduced with ^31^P NMR spectroscopy. A range of concentrations of free di-HMBP (NMR ^31^P{1H} (80.9 MHz): 19.17 ppm solution added with NaH_2_PO_4_ (in capillary, 10^−1^ mol · L^−1^; NMR ^31^P{1H} (80.9 MHz): 0 ppm) was prepared for calibration. The di-HMBP molecules are removed from magnetic *γ*Fe_2_O_3_ nanoparticles by addition of sodium hydroxide NaOH (1 mol · L^−1^) in order to avoid shifting of the ^31^P NMR signal. The supernatant is analyzed with ^31^P NMR and the concentration (number of molecules per nanocrystal) of di-HMBP into the sample is deduced from this calibration plot.

### 2.5. Analysis of the Size and Surface Charges of the *γ* *F* *e*_2_ *O*_3_@Di-HMBP Nanocrystals

The mean particle size was determined by transmission electron microscopy. Colloid suspensions were deposited directly onto a carbon-coated copper grid. The size and the zeta potential of the nanocomplex was determined by dynamic laser light scattering (DLS) on a Nano-ZS (Red Badge) ZEN 3600 device (Malvern Instruments, Malvern, UK. Each sample was analyzed at room temperature with diluted ferrofluid ([Fe] = 5 · 10^−4^ mol · L^−1^) at pH = 7.4.

### 2.6. Calcium Complexometric Titration

Standard procedures with Eriochrome black* T* (EBT) was used to quantify the amount of calcium ions in solution. The EBT was mixed to *γ*Fe_2_O_3_@di-HMBP (or free di-HMBP) aqueous solution ([Fe] = 1, 47 · 10^−2^ mol · L^−1^) at pH 10. Then this solution is titrated with calcium solution ([Ca^2+^] = 1, 44 · 10^−4^ mol · L^−1^) until the color solution change from blue to pink for free di-HMBP and from green to brown for *γ*Fe_2_O_3_@di-HMBP particles solution. The variation of color is due to the complexation between EBT and calcium ions. Then the amount of calcium ions complexed with the HMBP functionality is deduced.

### 2.7. Magnetic Properties and Magnetic Resonance Imaging

The magnetic behavior of the as-synthesized nanoparticles is characterized using the MIAplex^R^ reader (Magnisense). The MIAplex reader [13] measures the nonlinear response of the magnetic labels when they are exposed to a multi-frequency alternating magnetic field. This specific signature [14] is based on d^2^B(H)/dH^2^. 

MR imaging of the test tubes was performed using a 4.7 T MR scanner (Bruker). For measurements of T1 relaxation times, axial spin echo (SE) sequences were obtained with TR values of 10,000 ms as well as TE of 16 ms at 4.7 T. For measurements of T2* relaxation times, axial T2*-weighted SE images were obtained with a TR of 800 ms and TE of 6.4 ms at 4.7 T.

### 2.8. In Vitro Hydroxyapatite Targeting

The lyophilized hydroxyapatite [15] with a ratio Ca/P equal to 1,64. HA (10 mg/mL) was suspended in a 5 millimeter *γ*Fe_2_O_3_@di-HMBP sol 0,4 mg/mL (Fe = 5 · 10^−3^ M). Then nanoparticles are incubated and shaken with HA at 37°C during 24 hours. After filtration and water washing with a syringe filter with 0.45 *μ*m pore size, HA is resuspended in sol and lyophilized for infrared spectroscopy. The concentration of nanoparticles remained in the water suspension was measured by UV/VIS spectrophotometer at 350 and 480 nm for the calculation of the amount bound to HA.

### 2.9. Cell Viability

Human osteosarcoma cells (MG63) line was cultured in Dulbecco's modified Eagle's medium (DMEM, Invitrogen) supplemented with 10% calf serum. MG63 osteoblast-like cells used in the present study were obtained from the American Type Culture Collection (ATCC N° CRL 1427).

Cell viability was evaluated using the MTT (3-(4,5-Dimethylthiazol-2-yl)-2,5-diphenyltetrazolium bromide) assay at day 1, day 3, and day 5. Cells were seeded at a density of 20 × 10^3^ cells/well in 96-well flat-bottom plates (Falcon, Strasbourg, France) and incubated in complete culture medium for 1, 3, and 5 days. Then, medium was removed and replaced by 10% FCS-medium containing increasing concentrations *γ*Fe_2_O_3_@di-HMBP nanocrystals. After 1, 3, and 5 days of incubation, cells were washed with phosphate buffered saline (PBS, Invitrogen) and incubated with 0.1 mL of MTT (2 mg/mL, Sigma-Aldrich) for additional 4 hours at 37°C. The insoluble product was then dissolved by addition of DMSO (Sigma-Aldrich). Optical density was measured at 570 nm using a Labsystems Multiscan MS microplate reader. Each in vitro experiment was performed three times, with four wells per sample per experiment.

### 2.10. Cell Labelling

The labeling of living cells is evaluated using Prussian blue staining for *γ*Fe_2_O_3_@di-HMBPs nanocrystals. The principle of Prussian blue staining is that the ferric iron (Fe^3+^) in the presence of ferrocyanide ion is precipitated as the highly colored and highly water-insoluble complex, potassium ferric ferrocyanide, Prussian blue. The cells were cultivated for 24 hours in eight-well chamber slides in the presence or not of *γ*Fe_2_O_3_@di-HMBPs nanocrystals. The cells were then washed three times with PBS, fixed with acetone (10 minutes) and dried at room temperature for 20 mn. The attached cell monolayer was incubated with 5% potassium ferrocyanide (5 minutes), washed with PBS and then incubated again with solution containing 5% potassium ferrocyanide and 10% hydrochloric acid for 10 minutes and washed with distilled water three times. The iron particles in the cells were observed as blue dots using an optical microscope with phase contrast.

## 3. Results and Discussion

Nanoparticles functionalization plays a major role within nanotechnologies applications. Scheme 1 describes the procedure to design a new MRI nanoparticle for targeted drug delivery to bone. Small *γ*Fe_2_O_3_ nanocrystals were chosen for their superparamagnetic behavior and their high T2 contrast agent sensitivity for MRI. The 1,5-dihydroxy-1,5,5-tris-phosphono-pentyl-phosphonic acid (di-HMBPs) was chosen for the two HMBP functionalities: one HMBP moiety as anchoring agent for *γ*Fe_2_O_3_ surface and the second as targeting function due to strong affinity for bone. Our approach requires the two HMBP functions of the molecule to be separated by a short spacer, to avoid the nanoparticles anchoring with the two HMBP moieties, leading to nanoparticles aggregation and lost of the specific bone targeting.

Maghemite *γ*Fe_2_O_3_ nanocrystals were prepared as described previously [16] by soft chemistry. At the end of the synthesis, a solution of di-HMBP in water at pH 4 is added to the bare nanoparticle dispersion. The pH was then progressively increased to pH 7.4 by the addition of sodium hydroxide NaOH, thus achieving a stable dispersion of nanoparticles.

**Figure 1:**
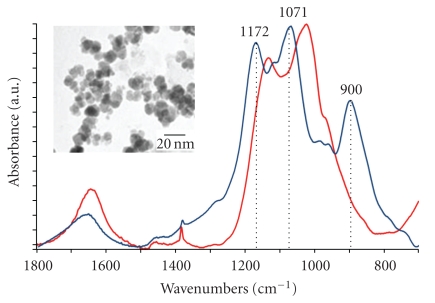
IR spectra of di-HMBP free molecules (blue curve) and *γ*Fe_2_O_3_@di-HMBP (red curve). Insert: transmission electron microscopy image taken of a nanoparticle solution at pH 7.

After dialysis, the dispersed solution is lyophilized. The powder is easily dispersed in water and the nanoparticles sols are stable over a broad range of pH (4–12) and concentration (over 40 wt%), in suitable ionic strength (<0.6 mol · L^−1^) and in various biological buffers such as PBS and Hepes. The TEM image (insert Figure 1) of deposited nanocrystals indicates an average diameter and a polydispersity, respectively, equal to 11 nm and 20%. 

IR spectroscopy analysis (Figure 1) shows that the phosphonate groups are highly interaction with the nanoparticle surface.

For the free HMBP-COOH molecules (blue curve), within the P–O stretching region (1200–900 cm^−1^), the spectrum exhibits two sharp peaks at 1172 and 900 cm^−1^, assigned to P*=*O and P–OH, respectively [17]. The broad band at 1071 cm^−1^ is characteristic for the vibrational mode for the PO_3_ group [18].

Comparing the *γ*Fe_2_O_3_@di-HMBP nanocrystals (red curve) with the free di-HMBP solution (blue curve), the large changes observed within the P–O stretching region (1200–900 cm^−1^) show that a strong interaction between the phosphonate headgroup and the Fe_2_O_3_ surface is present. These results are consistent with phosphonate binding to the oxide surface [19] and we can suggest that the Fe atoms within the particle surface are coordinated by oxygen atoms from the phosphonate groups [20].

^31^P NMR titration is used in order to quantify the average number of molecules per nanocrystal. An average number of 2100 ± 100 di-HMBP molecules per nanoparticle is obtained, corresponding to 0.1 equivalent per Fe ions (around 0.3 per surface Fe ions).

 Dynamic light scattering was used to characterize zeta potential and hydrodynamic diameter. This measurement is an indication of surface charge on a particulate species, which plays an important role in determining solution stability, susceptibility to aggregation and precipitation problems, as well as protein and cellular surface binding in vivo. At physiological pH, the *γ*Fe_2_O_3_@di-HMBPs particles exhibit a negative zeta potential (−54 mV) and a hydrodynamic diameter of 36 nm suggesting the presence of few aggregates (mean crystalline core of 11 nm). The negative charge surface suggests the presence of free HMBP functionalities on the magnetic core of the particle (Scheme 1). To determine the number of free HMBP, we used standard procedures of colorimetric tests to deduce the number of calcium ions complexed per *γ*Fe_2_O_3_@di-HMBP nanoparticles. For free di-HMBP molecules, we found 3.8 calcium ions complexed per molecule meaning that each HMBP functionality may complex about 2 calcium ions. The amount of calcium ions complexed per nanoparticle is found equal to 3100 ± 200. Considering that each HMBP functionality may complex 2 calcium ions, an amount of 1550 free HMBP per nanoparticle is deduced. This result is consistent with NMR measurements leading to 2100 HMBP per nanoparticle. Hence, the free HMBP functionalities at the outer of the nanoparticles surface should allow their bone targeting and the increase of bone mineral density.

The magnetic properties of these nanoparticles have been studied using a MIAplex^R^ reader. 

**Figure 2:**
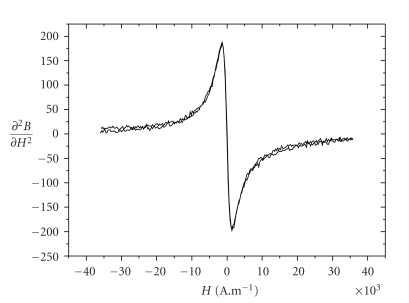
*γ*Fe_2_O_3_@di-HMBP second derivative of the magnetization recorded at pH 7.4, in H_2_O solutions.

The second derivative of magnetization d^2^B(H)/dH^2^ (Figure 2), presents one maxima and one minima with no hysteresis loop. This specific magnetic signature is characteristic of superparamagnetic behavior of particles with low dipolar interaction [21]. This superparamagnetic behavior allows to use these particles as contrast agent for MRI. 

**Figure 3:**
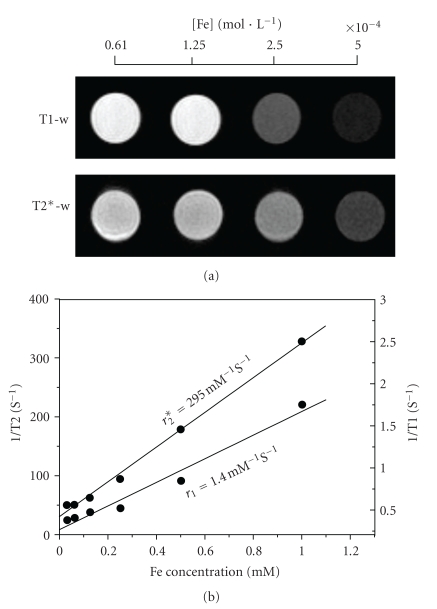
(a) T1 weight MR images and T2* weight MR images of aqueous solutions of as-synthesized nanoparticles at different Fe concentrations; (b) T1 and T2* relaxation rates (1/T1, 1/T2*) plotted against the Fe concentration for the various aqueous solutions.

To investigate the MR signal enhancement effects, the aqueous as-prepared nanoparticles at different Fe concentrations were measured on a 4.7 T MRI scanner. As shown in Figure 3(a), both T1 and T2* weighted images change drastically in signal intensity with an increasing amount of nanoparticles, indicating that as synthesized nanoparticles generated MR contrast on both longitudinal (T1) and transverse (T2*) proton relaxation times weighted sequences.  Figure 3(b) shows the relaxation rates 1/T1 and 1/T2* as a function of the iron concentration. The relaxation rates varied linearly with the iron concentration, as expected. The longitudinal *r*_1_ and transverse *r*_2_* relaxivities (corresponding to the slopes of the lines) are found to be 1.40 Fe mM^−1^s^−1^ and 295 Fe mM^−1^s^−1^, respectively. Such values for *r*_1_ and *r*_2_* suggest that HMBP coated nanoparticles can act as both T1 and T2* contrast agents taking into account their small size, but seem to be more favourable as T2* contrast agents due to their much larger *r*_2_* value. 

**Figure 4:**
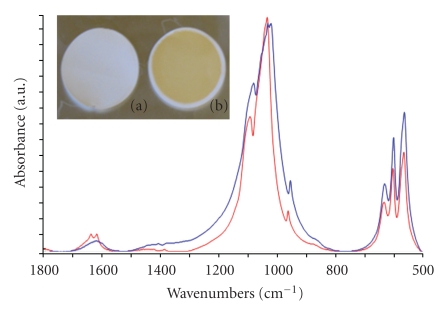
IR spectra of HA (red curve) and HA incubated with *γ*Fe_2_O_3_@di-HMBP (blue curve) for 24 hours and separated from free nanoparticules. Insert: optical image taken from HA (a) and HA incubated with *γ*Fe_2_O_3_@di-HMBP (b).

One of the factors that makes HMBP most potent BP drugs is its high skeletal uptake and retention, which is directly related to its affinity towards hydroxyapatite [22] (HA). To demonstrate the specific targeting of *γ*Fe_2_O_3_@di-HMPBs nanocrystals to bone, standard in vitro assay [12] were performed to demonstrate the strong affinity of those new MRI contrast agent with hydroxyapatite. A *γ*Fe_2_O_3_@diHMBP sol ([Fe] = 5 · 10^−3^ M) have been incubated with HA at 37°C, and then separated and washed using a 0.45 *μ*m filter. The binding capacity of the as-synthesized nanocomplexes has been studied using UV-vis and infrared (Figure 4) spectroscopies. As shown insert Figure 4, the change of HA color from white to brown indicates *γ*Fe_2_O_3_@di-HMBPs binds HA with very high affinity due to the high amount of iron nanoparticles within HA. The concentration of nanoparticles remained in the water suspension was measured by UV-vis spectrophotometer at 350 and 480 nm for the calculation of the amount bound to HA. The deduced bound amount is equal to 0.05 ± 0.01 mg of nanoparticles per mg of HA (eq. 0.19 mM HMBP per mg HA). Figure 4 displays the IR spectrum of HA (blue curve) and incubated HA with *γ*Fe_2_O_3_@diHMBP nanoparticles (red curve).

The HA spectrum (red curve) exhibits different bands between 1250–600 cm^−1^ that are characteristic of the P–O stretching region within HA [10]. For the HA nanocomplex, the analysis of the P–O stretching region is complicated due to strong background absorbance of the HA matrix (**ν**(PO4)) [23]. The HA incubated with *γ*Fe_2_O_3_@di-HMBP (blue curve), the P–O stretching region is broadened compared to initial HA. This is very difficult to clearly assign this effect. Obviously, more experiments are needed to elucidate the exact mechanism of nanoparticle surface bonding on HA.

 The magnemite nanocrystals deduced from UV-vis spectroscopy and the brown color (insert Figure 4) of incubated HA with *γ*Fe_2_O_3_@di-HMBP nanocrystals are suggesting selective interaction of the nanocomplex with HA and then potential targeting to bone. 

**Figure 5:**
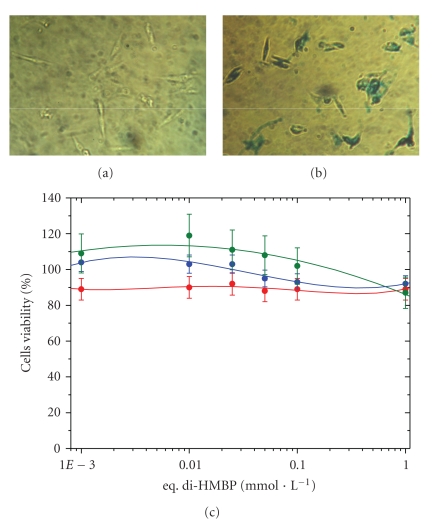
Optical blue prussian images of MG63 cells control (a) and MG63 cells incubating 24 hours with *γ*Fe_2_O_3_@di-HMBPs at 100 *μ*M (b). Comparative effects of *γ*Fe_2_O_3_@di-HMBPs on MG-63 osteoblast cells proliferation (c) for 24 hours, (red curve), 3 days (blue curve) and 5 days (green curve).

In order to assess cell viability we performed viability tests on osteosarcoma MG-63 cells, a cancer line, but a pertinent model to study efficiently the behavior of osteoblastic cell line [24]. The as-synthesized nanocrystals were incubated with MG63 osteosarcoma cells precultured for 24 hours, 3 days and 5 days for various extra cellular iron concentrations up to 3 mmol · L^−1^ (1 eq. mmol · L^−1^ di-HMBP). The proliferation of MG63 cells was indicated by the MTT assay as shown in Figure 5).

For the three times of incubation, MTT proliferation assay showed normal growth of osteoblast cells. No cytoxicity was observed. To determine the intracellular uptake of *γ*Fe_2_O_3_@di-HMBPs nanocrystals, blue prussian imaging was performed on human MG63 cells (Figures 5(a) and 5(b)). The iron particles into the cells were observed as blue dots using an optical microscope with phase contrast (Figure 5(b)). This picture indicates massive and uniform internalization of nanocrystals within the cells. Hence, the *γ*Fe_2_O_3_@di-HMBPs nanocrystals may act as a diagnostic and therapeutic system. A full biological study is in progress to understand the mechanism of such nanoparticles for osteoporosis treatment and diagnostic. The aim of this work is to describe influences of nanoparticles on protein expression patterns related to the differentiation and mineralization of bone-forming cells, viability, remodeling of cell architecture, cell adhesion, and assembly of extracellular matrix in human normal cells.

## 4. Conclusion

A bone-targeted MRI contrast agent have been designed with superparamagnetic nanoparticles and bisphosphonate moieties. HMBP functionalities exhibits highly iron and calcium complexing effects. To test feasibility of such nanosystem, this system have been complexed to hydroxyapatite to demonstrate bone targeting and increasing bone mineral density to reduce the incidence of major osteoporotic fracture. Moreover, the superparamagnetic behavior of such nanoparticle allows them to be used as MRI contrast agent in order to improve the therapeutic diagnostic for osteoporosis.
